# Association Between Lipids and Arterial Stiffness for Primary Cardiovascular Prevention in a General Middle-Aged European Population

**DOI:** 10.3389/fcvm.2022.899841

**Published:** 2022-05-27

**Authors:** Alexandre Vallée

**Affiliations:** Department of Epidemiology-Data-Biostatistics, Delegation of Clinical Research and Innovation (DRCI), Foch Hospital, Suresnes, France

**Keywords:** arterial stiffness, lipid, atherosclerosis, TG/HDL, cholesterol, lipid ratios, triglycerides, lipoprotein(a)

## Abstract

**Background:**

Dyslipidemia contributes to the progression of arterial stiffness (AS). The purpose of this study was to investigate the association of the different lipid parameters with arterial stiffness index (ASI) in a middle-aged population free of cardiovascular (CV) disease.

**Methods:**

Among 71,326 volunteers from the UK Biobank population, total cholesterol (TC), triglycerides (TG), low-density lipoprotein cholesterol (LDL), high-density lipoprotein cholesterol (HDL), lipoprotein (a) [Lp(a)], apolipoproteins A and B (Apo A and Apo B), and ASI were measured. Values for non-HDL, TC/HDL, TG/HDL, and LDL/HDL were calculated. AS was defined as an ASI > 10 m/s. Associations between lipid parameters and ASI were performed using multiple linear logistic regressions. The results reported from univariate models were the squared partial correlation coefficient, *r*^2^, and from multivariate models, the adjusted coefficient of determination, *R*^2^, to describe the contribution of ASI variability for each lipid parameter.

**Results:**

We found that TG/HDL was mainly associated with ASI [β = 0.53 (0.01), *r*^2^ = 3.66%, *p* < 0.001 and adjusted β = 0.21 (0.01), *R*^2^ = 13.58%, *p* < 0.001] and AS [odds ratio (OR) = 1.86 (1.80–1.92), *r*^2^ = 1.65%, *p* < 0.001 and adjusted OR = 1.15 (1.13–1.17), *R*^2^ = 8.54%, *p* < 0.001] rather than the others. TG/HDL remained the only lipid parameter that showed added value in linear multivariate models. TG/HDL remained less associated with AS than age (*r*^2^ = 5.55%, *p* < 0.001), mean blood pressure (BP; *r*^2^ = 5.31%, *p* < 0.001), and gender (*r*^2^ = 4.44%, *p* < 0.001), but more highly associated than body mass index (BMI; *r*^2^ = 1.95%, *p* < 0.001), heart rate (HR; *r*^2^ = 0.81%, *p* < 0.001), fasting glucose (*r*^2^ = 0.18%, *p* < 0.001), tobacco (*r*^2^ = 0.05%, *p* < 0.001), and glomerular filtration rate (GFR; *r*^2^ = 0.01%, *p* < 0.001).

**Conclusions:**

In primary CV prevention, lipids, especially through the TG/HDL ratio, could be more instructive in preventing the increase in AS than other modifiable factors.

## Introduction

Atherosclerosis is a major pathological mechanism of cardiovascular (CV) diseases (1), but early atherosclerosis lacks specific manifestations, thus it can easily go undetected in its early stages (2). With the gradual development of medical equipment and the continuous improvement of the approach to CV diseases, the assessment of arterial stiffness (AS) by noninvasive methods has become the aim of clinicians. AS, measured by the arterial stiffness index (ASI), can be considered a major denominator in target organ damage (3). The increase in AS leads to increased blood pressure (BP) and promotes vascular remodeling leading to atherosclerosis (4). Moreover, increased luminal pressure accelerates the formation of atheroma and stimulates the production of excessive collagen and its deposition in arterial walls, leading to atherosclerosis (5). Plaque formation and AS depend in part on the same pathophysiological mechanisms that cause the accumulation of an extracellular matrix of arterial walls (6). ASI measurement has been shown to be a marker of AS (7) which is the capacity of the arteries to expand and contract with a cardiac flow. AS can be an integrator of long-lasting arterial wall damage leading to luminal dilation due to an increase in collagen deposition (8). It is associated with coronary atherosclerosis (9), CV events (10), and inflammatory disorders (11).

Lipid parameters, including total cholesterol (TC), triglycerides (TG), low-density lipoprotein cholesterol (LDL), and high-density lipoprotein cholesterol (HDL), could have a main impact in CV risk events, and especially atherosclerosis (12). Furthermore, lowering LDL participates in the prevention and treatment of CV diseases (13–15). Recent European guidelines of the ESC highlighted the importance of lowering LDL in cases (16). Nevertheless, other lipid parameters, such as TC/HDL, TG/HDL, LDL/HDL ratios, and lipoprotein (a) [Lp(a)], apolipoproteins A and B (Apo A and Apo B) could improve the prediction of atherosclerosis and CV diseases (15, 17–19). Some studies have shown that these other parameters could be more informative than LDL alone for the prediction of atherosclerosis and CV risk events (20). Moreover, recent studies have shown that these lipid parameters could be associated mainly with AS rather than LDL (21, 22). The guidelines of the British National Institute for Health and Care Excellence guidelines and those of the American National Lipid Association recommend that other lipid parameters could be a better indicator than LDL (23). Nevertheless, few investigations have been performed for these lipid parameters in large populations of middle-aged participants without CV events for primary CV prevention. Thus, the purpose of this study was to investigate the association of the different lipid parameters with ASI in a middle-aged European population.

## Methods

### UK Biobank Population

The UK Biobank is a prospective cohort for the investigation, prevention, diagnosis, and treatment of chronic diseases, such as CV diseases in adults. A total of 502,478 Britons across 22 UK cities from the UK National Health Service Register were included between 2006 and 2010. The cohort was phenotyped and genotyped, by participants who responded to a questionnaire; a computer-assisted interview; physical and functional measures; and blood, urine, and saliva samples (24). Data included socioeconomic status, behavior and lifestyle, a mental health battery, clinical diagnoses and therapies, genetics, imaging, and physiological biomarkers from blood and urine samples. The cohort protocol can be found in the literature (25).

### Ethical Considerations

All participants provided electronic informed consent, and the UK Biobank received ethical approval from the North West Multi-center Research Ethics Committee (MREC) covering the whole of the UK. The study was conducted according to the guidelines of the Declaration of Helsinki, and was approved by the North West Haydock Research Ethics Committee (protocol code: 21/NW/0157, date of approval: 21 June 2021). For details: https://www.ukbiobank.ac.uk/learn-more-about-uk-biobank/about-us/ethics.

### BP Measurement

Systolic and diastolic blood pressure (SBP and DBP) were measured two times at the assessment center by using an automated BP device (Omron 705 IT electronic BP monitor; OMRON Healthcare Europe B.V. Kruisweg 577 2132 NA Hoofddorp), or manually by using a sphygmomanometer with an inflatable cuff in association with a stethoscope if the BP device failed to measure BP or if the largest inflatable cuff of the device did not fit around the individual's arm (26).

The participant was seated in a chair for all measurements. They were carried out by nurses trained in BP measurement (27). Multiple readings available for one participant were averaged. The Omron 705 IT BP monitor met the Association for the Advancement of Medical Instrumentation SP10 standard and was validated by the British Hypertension Society protocol, with an overall “A” grade for both SBP and DBP (28). Nevertheless, automated devices measure higher BP in comparison with manual sphygmomanometers, thus we adjusted both SBP and DBP, which were measured with the automated device using algorithms (29):

For SBP, we performed the following algorithm:


$$\begin{array}{l} \text{SBP = 3}.\text{3171+0}.\text{92019}\times SBP\;\left(\text{mmHg}\right)\text{+6}.\text{02468}\\ \;\times \text{ sex }(\text{male = 1};\text{female=}\;\text{0}). \end{array}$$

For DBP, we performed the following algorithm:


$$\begin{array}{l} \text{DBP}=\text{14}.\text{5647+0}.\text{80929}\times \text{DBP }\left(\text{mmHg}\right)\text{+2}.\text{01089}\\ \;\times \text{ sex }(\text{male=1};\text{female=}\;\text{0}). \end{array}$$

These adjusted BP values were used for all calculations, including mean BP calculation.

Mean BP was calculated as:


$$\text{mean BP=}\frac{\left(\text{SBP+2}\times \;\text{DBP}\right)}{\text{3}}.$$

### Outcomes

Pulse wave ASI was measured by a noninvasive method during a volunteer's visit to a UK Biobank Assessment Center. The pulse waveform was taken by clipping a photoplethysmographic transducer (PulseTrace PCA 2^TM^, CareFusion, USA) to the rested volunteer's finger (any finger or thumb, usually the index finger). Volunteers were asked to breathe in and out slowly five times in a relaxed fashion, and readings were taken over 10–15 s. ASI is performed from a single peripheral pulse waveform. The carotid-to-femoral pulse transit time was estimated from the dicrotic waveform as the time difference between a forward compound when the pressure is transmitted from the left ventricle to the finger and a reflected or backward compound as the wave is transmitted from the heart to the lower body *via* the aorta (30). ASI was estimated in meters per second (m/s) as H/PTT. H is the height of the individual, and PTT is the pulse transit time or the peak-to-peak time between the systolic and diastolic wave peaks in the dicrotic waveform (30). This methodology has been validated by comparing it with carotid-femoral pulse wave velocity (PWV). These studies concluded that both measurement methods were highly correlated to each other. ASI was a simple, operator-independent, nonexpensive, and rapid method (31–33). We excluded extreme outlier ASI values [defined as mean ± 5^*^standard deviation (SD)] from our analyses.

### Laboratory and Clinical Parameters

Current tobacco smokers were defined as participants who responded “yes, on most or all days” at the question “do you smoke tobacco now.” Body mass index was calculated as weight (in kg) divided by height^2^ (m). Biological parameters were detailed in the UK Biobank protocol (34).

Lipid parameters included in the analyses were: TC (mmol/L), TG (mmol/L), HDL (mmol/L), LDL (mmol/L), Apo A (mmol/L), Apo B (g/L), lipoproteins (a) (L(pa), nmol/L), ratios : LDL/HDL, TG/HDL, TC/HDL, and non-HDL (as TC—HDL).

For exclusion criteria: CV diseases were defined as heart attack, angina, and stroke, diagnosed by a doctor and reported in questionnaires. Medications (antihypertensives, statins, and antidiabetics) were characterized by the question: “Do you regularly take any of the following medications?”

### Study Population

Of the 502,478 volunteers from the UK Biobank, for the purposes of this study, we excluded participants with previous CV diseases, participants with medications (antihypertensive, statins, and antidiabetic medications), participants with severe hypertension (SBP ≥ 180 mmHg or DBP ≥ 110 mmHg), participants with severe obesity (BMI ≥ 40 kg/m^2^), participants with chronic kidney disease (CKD, defined by the calculated glomerular filtration rate (GFR) <60 ml/min/1.73 m^2^), participants with high values of LDL (LDL > 4.9 mmol/l), participants with high values of TG (TG > 8 mmol/l) according to SCORE (35), and extreme outlier ASI values and missing data for covariates. Therefore, we analyzed 71,326 volunteers.

### Statistical Analysis

The characteristics of the study population were described as means with SD for continuous variables. Categorical variables were described as numbers and proportions. Comparisons between groups were performed using Student's test or the Mann–Whitney test for continuous variables. Pearson's χ^2^ test was performed for categorical variables. AS was defined as ASI > 10 m/s. For each lipid parameter, the ability of logistic regression models to allow discrimination of ASI > 10 m/s was quantified by the area under the receiver operating characteristic (ROC) curve (AUC), which measures the ability of a classifier to distinguish between classes and is used as a summary of the ROC curve. The higher the AUC, the better the performance of the model in distinguishing between the two groups. The maximum Youden's index:


$$J=\max_{c}[S_{e}\left(c\right)+S_{p}\left(c\right)-1]$$

was chosen to determine the optimal thresholds (c) of lipid parameters for the discrimination of ASI > 10 m/s (as a definition of AS).

Univariate linear and logistic regressions were performed to assess the relationship between lipid parameters (as continuous values and thresholds) and continuous ASI values and AS status. Results reported for each model were the squared partial correlation coefficient, *r*^2^, which was used to describe the contribution of ASI variability for each lipid parameter.

Multiple linear and logistic regressions with adjustment for age, BMI, mean BP, heart rate (HR), gender, GFR, glucose, and tobacco status were performed to assess the independent relationship between lipid parameters (as continuous values and thresholds) and continuous ASI values and AS status. The results of multiple models would be the adjusted coefficient of determination, *R*^2^. One lipid parameter at a time was introduced into each model to avoid a collinear effect. The added values of the lipid parameters were performed by comparing of *R*^2^ of the adjusted models and the *R*^2^ of the adjusted models + each lipid parameter. Differences in correlation coefficients were assessed using Steiger's *Z* tests for comparison of correlations. Statistics were performed with SAS software (version 9.4; SAS Institute, Carry, NC, USA). The value of *p* < 0.05 was considered to indicate statistical significance.

## Results

The characteristics of the study population (*n* = 71,326) are described in Table 1 and divided into two groups [participants with ASI > 10 m/s, *n* = 24,363 (34.2%) and participants with ASI <10 m/s, *n* = 46,963 (65.8%)]. Participants with AS were older (*p* < 0.001) and presented higher levels of TC (*p* < 0.001), LDL (*p* < 0.001), HDL (*p* < 0.001), TG (*p* < 0.001), TC/HDL (*p* < 0.001), TG/HDL (*p* < 0.001), and LDL/HDL (*p* < 0.001) but there were no differences for Lp(a) (*p* = 0.768).

**Table 1:** Characteristics of the study population.

	**Overall Population** ***N #x0003D;*** **71,326**		**ASI > 10m/s** ***N =*** **24,363 (34.2%)**		**ASI < 10m/s** ***N =*** **46,963 (65.8%)**		***P-*value**
Age (years)	54.79	8.23	57.31	7.52	53.49	8.28	<0.001
Arterial stiffness index (ASI), m/s	9.05	2.90	12.33	1.99	7.35	1.51	<0.001
Body mass index (BMI), Kg/m2	26.36	4.03	27.02	3.95	26.0	4.02	<0.001
Glucose, mmol/L	4.95	(4.67–5.27)	4.97	(4.68–5.29)	4.94	(4.66–5.26)	<0.001
Lipoprotein (a), nmol/L	20.7	(9.5–60.6)	20.8	(9.6–59.9)	20.66	(9.5–60.9)	0.768
LDL cholesterol, mmol/L	3.58	0.66	3.66	0.64	3.53	0.67	<0.001
HDL cholesterol, mmol/L	1.49	0.37	1.44	0.36	1.53	0.37	<0.001
Total cholesterol, mmol/L	5.73	0.87	5.79	0.86	5.69	0.88	<0.001
Triglycerides, mmol/L	1.64	0.93	1.77	0.96	1.49	0.84	<0.001
Apolipoprotein A, g/L	1.56	0.27	1.53	0.26	1.58	0.27	<0.001
Apolipoprotein B, g/L	1.03	0.19	1.05	0.18	1.02	0.19	<0.001
TC/HDL	3.88	(3.25–4.65)	4.13	(3.45–4.91)	3.76	(3.18–4.51)	<0.001
TG/HDL	0.92	(0.60–1.49)	1.09	(0.69–1.73)	0.84	(0.56–1.35)	<0.001
LDL/HDL	2.54	0.79	2.70	0.81	2.46	0.77	<0.001
Non HDL, mmol/L	4.23	0.84	4.36	0.82	4.17	0.84	<0.001
Systolic blood pressure (SBP), mmHg	129.5	16.7	133.7	16.1	127.3	16.5	<0.001
Diastolic blood pressure (DBP), mmHg	80.9	8.1	83.2	7.9	79.8	7.9	<0.001
Mean blood pressure (MBP), mmHg	97.1	10.3	100.0	9.89	95.6	10.11	<0.001
Heart rate (HR), bpm	67.8	10.37	68.6	10.2	67.4	10.4	<0.001
Glomerular filtration rate (GFR), mL/min/1.73 m^2^	91.72	(82.14–102.71)	91.15	(81.52–102.08)	93.6	16.0	<0.001
Gender (women)	40459	56.72%	10912	44.79%	29547	62.92%	<0.001
Current tobacco	5198	7.29%	2346	9.63%	2852	6.07%	<0.001

*Mean and standard deviation (SD) or median and (25–75th percentile) for continuous variables and n and percentage for categorical variables*.

Thresholds of lipid parameters were performed for AS status (Table 2). Nevertheless, the accuracies of the models remained low.

**Table 2:** Determination of thresholds for each of the lipid parameters by continuous arterial stiffness index (ASI) values.

**Parameters**	**Threshold**	***P-*value**	**AUC**
Total cholesterol, mmol/L	5.28	<0.001	0.534
Triglycerides, mmol/L	1.269	<0.001	0.598
HDL cholesterol, mmol/L	1.413	<0.001	0.574
LDL cholesterol, mmol/L	3.464	<0.001	0.552
NonHDL	4.143	<0.001	0.567
TC/HDL	3.88835	<0.001	0.595
TG/HDL	0.89532	<0.001	0.602
LDL/HDL	2.53109	<0.001	0.591
Apolipoprotein A1, g/L	1.510	<0.001	0.551
Apolipoprotein B, g/L	1.021	<0.001	0.564
Lipoprotein (a), nmol/L	NS	0.768	NS

*NS: non-significant*.

### Univariate Linear and Logistic Analyses of Lipid Parameters and ASI

All lipid parameters (in continuous values and in thresholds) showed a significant correlation with continuous ASI (for all, *p* < 0.001), except Lp(a) (*p* = 0.773 and *p* = 0.381, respectively). The squared correlation coefficients (*r*^2^) showed low levels for the relationship between lipid parameters and ASI (*r*^2^ = 0.36–3.67% for continuous ASI and *r*^2^ = 0.37–3.05% for AS, Table 3). The highest relationship with continuous ASI was observed for TG/HDL, which showed a significantly higher correlation than all other lipid parameters (*r*^2^ = 3.67% for continuous ASI and *r*^2^ = 3.05% for AS) (Table 3).

**Table 3:** Univariate linear regression models for continuous ASI values with each lipid parameter in continuous values and in threshold cutoffs.

**Continuous values**	**Beta (SE)**	***P-*value**	***R* ^2^**	*P-*value^*^	**Threshold values**	**Beta (SE)**	***P-*value**	***r* ^2^**	*P-*value^*^
Total cholesterol, mmol/L	0.20 (0.01)	<0.001	0.003642	<0.001	Total cholesterol, mmol/L	0.19 (0.01)	<0.001	0.003769	<0.001
Triglycerides, mmol/L	0.56 (0.01)	<0.001	0.029211	<0.001	Triglycerides, mmol/L	0.48 (0.01)	<0.001	0.017061	<0.001
HDL cholesterol, mmol/L	−1.12 (0.03)	<0.001	0.020650	<0.001	HDL cholesterol, mmol/L	−0.39 (0.01)	<0.001	0.017618	<0.001
LDL cholesterol, mmol/L	0.43 (0.02)	<0.001	0.009424	<0.001	LDL cholesterol, mmol/L	0.26 (0.01)	<0.001	0.007679	<0.001
NonHDL	0.44 (0.01)	<0.001	0.016009	<0.001	NonHDL	0.31 (0.01)	<0.001	0.011473	<0.001
TC/HDL	0.52 (0.01)	<0.001	0.032794	0.020	TC/HDL	0.47 (0.01)	<0.001	0.025767	0.004
TG/HDL	0.53 (0.01)	<0.001	0.036647	Ref.	TG/HDL	0.51 (0.01)	<0.001	0.030522	Ref.
LDL/HDL	0.65 (0.01)	<0.001	0.031383	0.001	LDL/HDL	0.45 (0.01)	<0.001	0.024226	<0.001
Apolipoprotein A1, g/L	−1.09 (0.04)	<0.001	0.010301	<0.001	Apolipoprotein A1, g/L	−0.26 (0.01)	<0.001	0.008049	<0.001
Apolipoprotein B, g/L	1.82 (0.06)	<0.001	0.014401	<0.001	Apolipoprotein B, g/L	0.30 (0.01)	<0.001	0.010733	<0.001
Lipoprotein (a), nmol/L	0.01 (0.01)	0.773	NS	NA	Lipoprotein (a), nmol/L	NS	0.01 (0.01)	0.381	nA

* *For comparison of r^2^ of lipid parameters with the apparent higher r^2^ as a reference, i.e., the model that includes the triglycerides/high-density lipoprotein cholesterol (TG/HDL) ratio*.

When studying AS status, TG/HDL remained the lipid parameter with the highest accuracy (as in the continuous value, *r*^2^ = 1.84% and as in the threshold, *r*^2^ = 1.65%) but did not present significant differences with continuous TG (*r*^2^ = 1.55%, *p* = 0.080), continuous TC/HDL (*r*^2^ = 1.56%, *p* = 0.090), and continuous LDL/HDL (*r*^2^ = 1.72%, *p* = 0.469) and did not present significant differences with the TG threshold (*r*^2^ = 1.51%, *p* = 0.398), the TC/HDL threshold (*r*^2^ = 1.45%, *p* = 0.228), and the LDL/HDL threshold (*r*^2^ = 1.37%, *p* = 0.090) (Table 4).

**Table 4:** Univariate logistic regression models for arterial stiffness (defined as ASI > 10 m/s) with each lipid parameter in continuous values and threshold cutoffs.

**Continuous**	**OR 95%CI**	***r* ^2^**	***P-*value**	*P-*value^*^	**Threshold**	**OR 95%CI**	***P-*value**	***r* ^2^**	*P-*value^*^
Total cholesterol, mmol/L	1.14 (1.12–1.16)	0.0024	<0.001	<0.001	Total cholesterol, mmol/L	1.30 (1.26–1.35)-	<0.001	0.0026	<0.001
Triglycerides, mmol/L	1.39 (1.37–1.41)	0.0155	<0.001	0.080	Triglycerides, mmol/L	1.82 (1.76–1.87)	<0.001	0.0151	0.398
HDL cholesterol, mmol/L	0.50 (0.48–0.52)	0.0110	<0.001	<0.001	HDL cholesterol, mmol/L	0.64 (0.62–0.66)	<0.001	0.0089	<0.001
LDL cholesterol, mmol/L	1.31 (1.28–1.35)	0.0056	<0.001	<0.001	LDL cholesterol, mmol/L	1.40 (1.35–1.44)	<0.001	0.0047	<0.001
NonHDL	1.32 (1.29–1.35)	0.0094	<0.001	<0.001	NonHDL	1.50 (1.45–1.55)	<0.001	0.0070	<0.001
TC/HDL	1.37 (1.35–1.39)	0.0156	<0.001	0.091	TC/HDL	1.78 (1.73–1.84)	<0.001	0.0145	0.228
TG/HDL	1.37 (1.34–1.39)	0.0184	<0.001	Ref.	TG/HDL	1.86 (1.80–1.92)	<0.001	0.0165	Ref.
LDL/HDL	1.49 (1.46–1.52)	0.0172	<0.001	0.469	LDL/HDL	1.75 (1.69–1.79)	<0.001	0.0137	0.090
Apolipoprotein A1, g/L	0.53 (0.50–0.56)	0.0051	<0.001	<0.001	Apolipoprotein A1, g/L	0.74 (0.72–0.76)	<0.001	0.0038	<0.001
Apolipoprotein B, g/L	3.23 (2.97–3.51)	0.0087	<0.001	<0.001	Apolipoprotein B, g/L	1.49 (1.44–1.54)	<0.001	0.0068	<0.001
Lipoprotein (a), nmol/L	1.00 (0.99–1.01)	NS	0.768	-	Lipoprotein (a), nmol/L	1.01 (0.98–1.05)	0.231	NS	-

*NA, not applicable; NS, nonsignificant.*

* *For comparison of r^2^ of lipid parameters with the apparently higher r^2^ as a reference, i.e., the model that includes TG/HDL*.

### Multiple Linear and Logistic Analyses of Lipid Parameters and ASI

Tables 5, 6 present the multiple linear and logistic regression analyses performed to assess the association between lipid parameters and ASI. Adjustment was performed for possible confounders, such as age, BMI, mean BP, tobacco status, GFR, fasting glucose, and gender.

**Table 5:** Multiple linear regression models for continuous ASI values with each lipid parameter in continuous values and in threshold cutoffs.

**Continuous values**	**Beta (SE)**	***P-*value**	***R* ^2^**	*P-*value^*^	**Threshold values**	**Beta (SE)**	***P-*value**	***R* ^2^**	*P-*value^*^
Total cholesterol, mmol/L	0.02 (0.01)	0.048	0.132154	0.028	Total cholesterol, mmol/L	0.02 (0.01)	0.048	0.132185	0.027
Triglycerides, mmol/L	0.21(0.01)	<0.001	0.134502	0.440	Triglycerides, mmol/L	0.17 (0.01)	<0.001	0.135089	0.658
HDL cholesterol, mmol/L	−0.49 (0.03)	<0.001	0.131917	0.019	HDL cholesterol, mmol/L	−0.16 (0.01)	<0.001	0.132379	0.036
LDL cholesterol, mmol/L	0.04 (0.01)	0.031	0.132210	0.030	LDL cholesterol, mmol/L	0.03 (0.01)	0.002	0.132254	0.030
Non HDL	0.07 (0.01)	<0.001	0.132544	0.049	Non HDL	0.05 (0.01)	<0.001	0.132441	0.040
TC/HDL	0.18 (0.01)	<0.001	0.135064	0.667	TC/HDL	0.14 (0.01)	<0.001	0.134135	0.306
TG/HDL	0.21 (0.01)	<0.001	0.135770	Ref.	TG/HDL	0.20 (0.01)	<0.001	0.135815	Ref.
LDL/HDL	0.21 (0.01)	<0.001	0.134584	0.470	LDL/HDL	0.14 (0.01)	<0.001	0.133982	0.264
Apolipoprotein A1, g/L	−0.42 (0.04)	<0.001	0.131293	0.006	Apolipoprotein A1, g/L	−0.08 (0.01)	<0.001	0.132546	0.046
Apolipoprotein B, g/L	0.29 (0.02)	<0.001	0.131491	0.009	Apolipoprotein B, g/L	0.05 (0.01)	<0.001	0.132456	0.041
Lipoprotein (a), nmol/L	0.01 (0.01)	0.385	NS	NA	Lipoprotein (a), nmol/L	0.01 (0.01)	0.885	NS	NA

*Adjustment was performed on age, gender, BMI, body mass index; BP, mean blood pressure; HR, heart rate; GFR, glomerular filtration rate; tobacco, and glucose. NA, not applicable; NS, non-significant.*

* *For comparison of R^2^ of lipid parameters with the apparently higher R^2^ as a reference, i.e., the model that includes TG/HDL*.

**Table 6:** Multiple logistic regression models for arterial stiffness (defined as ASI > 10 m/s) with each lipid parameter in continuous values and in threshold cutoffs.

**Continuous values**	**OR 95% CI**	***P-*value**	***R* ^2^**	*P-*value^*^	**Threshold values**	**OR 95% CI**	***P-*value**	***R* ^2^**	*P-*value^*^
Total cholesterol, mmol/L	1.01 (0.99–1.03)	0.460	NS	NA	Total cholesterol, mmol/L	1.05 (1.02–1.09)	0.006	0.0831	0.164
Triglycerides, mmol/L	1.15 (1.13–1.17)	<0.001	0.0853	0.952	Triglycerides, mmol/L	1.28 (1.24–1.33)	<0.001	0.0850	0.548
HDL cholesterol, mmol/L	0.70 (0.66–0.74)	<0.001	0.0849	0.762	HDL cholesterol, mmol/L	0.81 (0.78–0.84)	<0.001	0.0844	0.529
LDL cholesterol, mmol/L	1.04 (1.01–1.06)	<0.001	0.0831	0.164	LDL cholesterol, mmol/L	1.06 (1.03–1.10)	<0.001	0.0831	0.164
NonHDL	1.06 (1.04–1.09)	<0.001	0.0834	0.226	NonHDL	1.10 (1.06–1.14)	<0.001	0.0833	0.204
TC/HDL	1.14 (1.12–1.16)	<0.001	0.0851	0.856	TC/HDL	1.24 (1.20–1.29)	<0.001	0.0845	0.586
TG/HDL	1.15 (1.13–1.17)	<0.001	0.0854	Ref.	TG/HDL	1.31 (1.26–1.35)	<0.001	0.0853	Ref.
LDL/HDL	1.17 (1.14–1.20)	<0.001	0.0848	0.716	LDL/HDL	1.23 (1.19–1.28)	<0.001	0.0844	0.545
Apolipoprotein A1, g/L	0.74 (0.69–0.80)	<0.001	0.0837	0.303	Apolipoprotein A1, g/L	0.89 (0.86–0.92)	<0.001	0.0834	0.226
Apolipoprotein B, g/L	1.29 (1.19–1.42)	<0.001	0.0833	0.204	Apolipoprotein B, g/L	1.10 (1.07–1.14)	<0.001	0.0833	0.204
Lipoprotein (a), nmol/L	1.00 (0.99–1.01)	0.691	NS	NA	Lipoprotein (a), nmol/L	1.00 (0.96–1.03)	0.941	NS	NA

*Adjustment was performed on age, gender, BMI, mean BP, HR, GFR, tobacco, and glucose. NA, not applicable; NS, nonsignificant.*

* *For comparison of R^2^ of lipid parameters with the apparently higher R^2^ as a reference, i.e., the model that includes TG/HDL*.

When considering continuous ASI values (Table 5), after adjustment, all the lipid parameters remained significant (*p* < 0.05) except Lp(a) (*p* = 0.385 and *p* = 0.885). The highest independent correlation with continuous ASI was observed for continuous TG/HDL (*R*^2^ = 13.58%) but without significant differences with continuous TG (*R*^2^ = 13.45%, *p* = 0.440), continuous TC/HDL (*R*^2^ = 13.51%, *p* = 0.667), and continuous LDL/HDL (*R*^2^ = 13.46%, *p* = 0.470). The same results were observed when considering the TG/HDL threshold (*R*^2^ = 13.58%) with the TG threshold (*R*^2^ = 13.51%, *p* = 0.658), the TC/HDL threshold (*R*^2^ = 13.41%, *p* = 0.306), and the LDL/HDL threshold (*R*^2^ = 13.40%, *p* = 0.264).

When considering AS status (Table 6), after adjustment, all lipid parameters remained significant (*p* < 0.001) except Lp(a) and TC. The highest independent correlation with AS status was observed for continuous TG/HDL (*R*^2^ = 8.54%) but without significant differences with all other lipid parameters. The same results were observed with the TG/HDL threshold (*R*^2^ = 8.53%), which did not present significant differences with all other lipid parameters.

When considering LDL as a reference, between TC (*p* = 0.973), TG (*p* = 0.163), HDL (*p* = 0.858), non-HDL (*p* = 0.839), TC/HDL (*p* = 0.082), and LDL/HDL (*p* = 0.148), only TG/HDL (*p* = 0.030) showed a significantly higher association in multivariate analyses with AS and continuous values compared to LDL.

Table 7 shows the multiple regression results of the (linear) associations between continuous ASI with continuous TG/HDL or TG/HDL threshold and confounding variables, and the (logistic) association between AS status with continuous TG/HDL or TG/HDL threshold and confounding factors. Continuous TG/HDL showed a significant added value for multiple linear models for continuous ASI values when adding it in the model (*R*^2^ 13.577% vs. *R*^2^ = 13.211%, respectively, *p* = 0.028). The same results were observed when the TG/HDL threshold was added (*R*^2^ = 13.582% vs. *R*^2^ = 13.211%, respectively, *p* = 0.026). However, for logistic regression models for AS, TG/HDL did not show a significant added value (*p* = 0.146 for continuous TG/HDL and *p* = 0.164 for the TG/HDL threshold).

**Table 7:** Multiple linear (model 1 for continuous ASI values) and logistic (model 2 for arterial stiffness) regression models with TG/HDL parameters in continuous values and in threshold cutoffs and with age, gender, BMI, mean BP, HR, GFR, tobacco, and glucose.

**Parameters**	**Beta (SE)**	***P-*value**	***R* ^2^**	***r* ^2^**	*P-*value^**^	**Parameters**	**Beta (SE)**	***P-*value**	***R* ^2^**	***r* ^2^**	*P-*value^**^
**Models 1: ASI continuous values**
Age (years)	0.08 (0.01)	<0.001	0.132153	0.055106	<0.001	Age (years)	0.08 (0.01)	<0.001	0.132153	0.055106	<0.001
Body mass index (BMI), Kg/m2	0.05 (0.01)	<0.001		0.019468	<0.001	Body mass index (BMI), Kg/m2	0.05 (0.01)	<0.001		0.019468	<0.001
Heart rate (HR), bpm	0.02 (0.01)	<0.001		0.008095	<0.001	Heart rate (HR), bpm	0.02 (0.01)	<0.001		0.008095	<0.001
Mean blood pressure (MBP), mmHg	0.03 (0.01)	<0.001		0.053076	<0.001	Mean blood pressure (MBP), mmHg	0.03 (0.01)	<0.001		0.053076	<0.001
Gender (men)	0.45 (0.01)	<0.001		0.044390	<0.001	Gender (men)	0.45 (0.01)	<0.001		0.044390	<0.001
Glucose, mmol/L	−0.06 (0.01)	<0.001		0.001763	<0.001	Glucose, mmol/L	−0.06 (0.01)	<0.001		0.001763	<0.001
Current tobacco	0.38 (0.02)	<0.001		0.005001	<0.001	Current tobacco	0.38 (0.02)	<0.001		0.005001	<0.001
GFR	0.01 (0.01)	0.012		0.000713	<0.001	GFR	0.01 (0.01)	0.012		0.000713	<0.001
TG/HDL (continuous)	0.21 (0.01)	<0.001	0.13577	0.036647	Ref.	TG/HDL (threshold)	0.20 (0.01)	<0.001	0.135815	0.030522	Ref.
			*P* = 0.028^*^						*P* = 0.026^*^		
**Parameters**	**OR 95% CI**	***P-*** **value**	***R* ^2^**	***r* ^2^**	***P-*** value^**^	**Parameters**	**OR 95% CI**	***P-*** **value**	***R* ^2^**	***r* ^2^**	***P-*** value^**^
**Models 2: Arterial stiffness status**
Age (years)	1.06 (1.05–1.07)	<0.001	0.0830	0.0387	<0.001	Age	1.06 (1.05–1.07)	<0.001	0.0830	0.0387	<0.001
Body Mass index (BMI), Kg/m2	1.04 (1.03–1.05)	<0.001		0.0106	<0.001	Body Mass index (BMI), Kg/m2	1.04 (1.03–1.05)	<0.001		0.0106	<0.001
Heart Rate (HR), bpm	1.02 (1.01–1.03)	<0.001		0.0021	<0.001	Heart Rate (HR), bpm	1.02 (1.01–1.03)	<0.001		0.0021	<0.001
Mean Blood Pressure (MBP), mmHg	1.02 (1.01–1.03)	<0.001		0.0324	<0.001	Mean Blood Pressure (MBP), mmHg	1.02 (1.01–1.03)	<0.001		0.0324	<0.001
Gender (men)	1.71 (1.65–1.77)	<0.001		0.0234	<0.001	Gender (men)	1.73 (1.67–1.79)	<0.001		0.0234	<0.001
Glucose, mmol/L	0.96 (0.94–0.98)	<0.001		0.0008	<0.001	Glucose, mmol/L	0.96 (0.94–0.98)	0.001		0.0008	<0.001
Current tobacco	1.79 (1.69–1.90)	<0.001		0.0032	<0.001	Current tobacco	1.80 (1.70–1.90)	<0.001		0.003	<0.001
GFR	1.02 (1.01–1.03)	0.002		0.0005	<0.001	GFR	1.02 (1.01–1.03)	0.004		0.005	<0.001
TG/HDL (continuous)	1.15 (1.13–1.17)	<0.001	0.0854	0.0184	Ref.	TG/HDL (threshold)	1.31 (1.26–1.35)	<0.001	0.0853	0.0165	Ref.
			P=0.146^*^						P=0.164^*^		

* *For comparison of R^2^ of adjusted model and the R^2^ of adjusted model + TG/HDL.*

** *For comparison of r^2^ of TG/HDL as a reference and the r^2^ of the other covariates*.

The other three lipid parameters (TG, TC/HDL, and LDL/HDL) that did not show significant differences with TG/HDL in multivariate analyses did not show significant added values: TC/HDL did not show added values for both linear models with continuous ASI values (*p* = 0.076 for continuous TC/HDL and *p* = 0.227 for the TC/HDL threshold). The same results were observed for TG (in linear regression models with continuous TG (*p* = 0.061) and with the TG threshold (*p* = 0.074) and for LDL/HDL (in linear regression models with continuous LDL/HDL (*p* = 0.139) and with the LDL/HDL threshold (*p* = 0.265). No added values were observed for both continuous and thresholds of lipid parameters in logistic regression models for AS status (data not shown).

In all multivariate models, TG/HDL showed a higher relationship with AS than BMI (*p* < 0.001), HR (*p* < 0.001), fasting glucose (*p* = 0.001), tobacco status (*p* < 0.001), and GFR (*p* = 0.004), but a lower relationship with AS than age (*p* < 0.001), mean BP (*p* < 0.001), and gender (*p* < 0.001) (Table 7).

## Discussion

The results of this study showed that the lipid parameter TG/HDL was more often associated with ASI and AS than the other lipid parameters. No significant differences between TG/HDL and TC/HDL, LDL/HDL and TG were observed in all models, but TG/HDL remained the only parameter that showed added value in multivariate models and when compared with the traditional LDL parameter TG/HDL remained the only significant parameter. To date, the TG/HDL ratio has been increasingly recognized as the main index in atherogenic particles, i.e., coronary artery disease (36). Under the effect of cholesterol ester transfer protein, high levels of TG can correlate with more active lipid exchanges. This results in increased LDL concentration and decreased HDL levels, enhancing atherosclerosis-inducing factors (37). TG/HDL could indicate the imbalance between atherogenic and protective lipoproteins and has shown predictive interest in assessing the extent of early-stage atherosclerosis (22). In contrast, the only lipid parameter, which remained nonsignificant in all models with AS, was Lp(a). No lipid parameter showed added values in the multivariate models, but all remained independently significant when AS status was considered. Nevertheless, in both linear and logistic models, TG/HDL showed a stronger relationship with AS than BMI, HR, fasting glucose, tobacco, and GFR but a weaker relationship than nonmodifiable factors, such as age, gender, and mean BP.

### AS and Lipid Parameters

Several investigations have shown positive associations between AS and lipid parameters (21, 22, 38–43). In specific populations, the same associations have been observed in both young and elderly participants (22, 44). Moreover, epidemiological findings have shown that lipid ratios (such as TG/HDL, TC/HDL, and LDL/HDL) were more often associated with CV risks than with single lipid parameters (22, 45). According to previous studies, the TG/HDL ratio was recognized as a major index of atherogenic particles by showing a stronger relationship with AS than other lipid parameters (21, 22, 36, 46, 47). Moreover, the TG/HDL ratio was mainly associated with predicting the incidence of coronary artery disease and CV mortality (48). Our study shows that TG/HDL was more often associated than other lipid parameters (such as isolated lipids and other lipid ratios) with ASI in a middle-aged population free of CV disease. This result was consistent with other studies in young, healthy populations (22, 46). Lp(a) was not associated with AS in our study, in concordance with other recent studies (49). To date, the relationship between Lp(a) and CV disease remains unclear even if it has been considered an atherosclerotic risk factor (17). The possible mechanisms linking Lp(a) to AS remain hypothetical. The physiological role of Lp(a) remains unknown, even if its pathogenic mechanisms have been investigated and have shown proatherogenic and inflammatory actions. Nevertheless, the relationship between Lp(a) and AS was little explored, except in elderly populations (50) or in specific hypertensive populations (51).

### TG/HDL and AS

The increase in TG has been associated with the production of reactive oxygen species (ROS) and the induction of insulin resistance. Moreover, TG leads to an increase in LDL particles responsible for atherogenic pathways (52) and to stimulation of arterial smooth muscles and dysfunction in endothelial vasodilatation (53). In parallel, HDL has been considered as an antiatherogenic lipoprotein, which activates the efflux of cellular cholesterol and reverses cholesterol to the liver (54). HDL is able to protect the body against atherosclerosis by downregulating the oxidation of lipoproteins and degrading activated oxidized phospholipids (55). Thus, the TG/HDL ratio could be of interest in the atherosclerosis pathway by showing the imbalance between atherogenic and protective lipoproteins. Moreover, previous findings have shown that the TG/HDL ratio has a high association with insulin resistance in individuals free from metabolic syndrome (56). Insulin resistance is associated with increased production of TG and decreased HDL levels (57). This relationship has been observed in overweight individuals with normal glucose tolerance (58).

### TC/HDL and LDL/HDL With the AS Index

In the different models, we found that TC/HDL and LDL/HDL did not show significant differences with TG/HDL but had no added value in the multivariate models. Moreover, in comparison with the isolated LDL parameter, these parameters remained nonsignificant unlike TG/HDL. As observed in previous studies, these parameters showed a significant relationship with AS (21, 46, 59). However, the findings observed could be the result of a decrease in the antiatherogenic constituent of the denominator (i.e., HDL) (60). The numerators TC and LDL showed a lower relationship with AS than TG. This can be explained by the possible lower relationship of TC/HDL and LDL/HDL in comparison with the TG/HDL ratio and their non-difference with LDL.

### Risk Factors and AS

In overall models, all cofounding factors remained significantly associated with AS, such as age, BMI, HR, mean BP, gender, fasting glucose, GFR, and tobacco status. These results were consistent with the literature (61). One main interesting point was the added value of TG/HDL compared to these factors. Indeed, while TG/HDL remained less associated with AS than age, mean BP, and gender, this ratio showed a significant and greater association than BMI, HR, fasting glucose, GFR, and tobacco. In primary CV prevention, lipids could be more informative in preventing the increase in AS than other modifiable factors.

### Strengths and Limitations

The main strength of this study is the very large sample size of the cohort. The cross-sectional observational design limits the relationship of causality. Reverse causation cannot be ruled out. A potential limitation could stem from the utilization of the Pulse trace device to measure AS on account of greater variability in ASI values relative to other available devices (62). The UK Biobank study showed a low response rate of 5.5% and possible volunteer bias might be involved. Nevertheless, given the large sample size and high internal validity, these are unlikely to affect the reported associations (63, 64). In addition, the study cohort consisted of middle-aged European participants, so our findings might not be generalized to other age groups and ethnic populations. Nevertheless, the UK Biobank used standardized protocols to collect anthropometric data; this ensures replication of data collection for all volunteers regardless of when, where, and by whom they are performed and adds validity to our results.

## Conclusions

We found a positive association between lipid parameters and AS. Our results showed that the TG/HDL ratio presented a stronger association than other conventional lipid parameters and lipid ratios in our middle-aged population free of CV disease. This TG/HDL ratio was easy to calculate and could participate in the prevention of the reduction of AS in clinical practice. Lp(a) remained the only lipid parameter not associated with AS in our study population. Moreover, longitudinal studies should be implemented to better investigate this association.

## Data Availability Statement

The original contributions presented in the study are included in the article/supplementary material, further inquiries can be directed to the corresponding author.

## Ethics Statement

The studies involving human participants were reviewed and approved by North West—Haydock Research Ethics Committee (Protocol Code: 21/NW/0157, date of approval: 21 June 2021). For details: https://www.ukbiobank.ac.uk/learn-more-about-uk-biobank/about-us/ethics. The patients/participants provided their written informed consent to participate in this study.

## Author Contributions

AV: conceptualization, methodology, formal analysis, and writing—original draft preparation.

## Conflict of Interest

The author declares that the research was conducted in the absence of any commercial or financial relationships that could be construed as a potential conflict of interest.

## Publisher's Note

All claims expressed in this article are solely those of the authors and do not necessarily represent those of their affiliated organizations, or those of the publisher, the editors and the reviewers. Any product that may be evaluated in this article, or claim that may be made by its manufacturer, is not guaranteed or endorsed by the publisher.

## Abbreviations

Apo A: Apolipoproteins A
Apo B: Apolipoproteins B
AS: arterial stiffness
ASI: arterial stiffness index
BP: blood pressure
CV: cardiovascular
DBP: diastolic blood pressure
HLD: high-density lipoprotein cholesterol
LDL: low-density lipoprotein cholesterol
LDL/HDL: ratio low-density lipoprotein cholesterol / high-density lipoprotein cholesterol
Lp(a): lipoprotein (a)
Non-HDL: total cholesterol—high-density lipoprotein cholesterol
SBP: systolic blood pressure
TC: total cholesterol
TC/HDL: ratio total cholesterol/high-density lipoprotein cholesterol
TG: Triglycerides
TG/HDL: ratio triglycerides/high-density lipoprotein cholesterol.
